# Cognitive Dysfunction Among People Living with HIV/AIDs: A Multi‐Center Cross‐Sectional Study from South‐West Nigeria

**DOI:** 10.1002/alz70857_098975

**Published:** 2025-12-24

**Authors:** Mayowa T Ogunronbi, Oladotun V Olalusi, Gabriel O Ogunde, Rufus O. Akinyemi

**Affiliations:** ^1^ Institute of Advanced Medical Research and Training (IAMRAT), College of Medicine, University of Ibadan, Ibadan Nigeria, Oyo, Nigeria; ^2^ Institute for Advanced Medical Research and Training, College of Medicine, University of Ibadan, Ibadan, Oyo State, Nigeria; ^3^ Institute for Advanced Medical Research and Training, College of Medicine, University of Ibadan, Ibadan, Oyo, Nigeria; ^4^ College of Medicine, University of Ibadan, Ibadan, Oyo State, Nigeria; ^5^ Institute of Advanced Medical Research and Training, College of Medicine, University of Ibadan, Ibadan, Oyo State, Nigeria; ^6^ Africa Dementia Consortium, Ibadan, Ibadan, Nigeria

## Abstract

**Background:**

Sub‐Saharan Africa (SSA) is witnessing a double burden of infectious and non‐communicable diseases, chief among which is HIV/AIDS and cognitive dysfunction. Besides population ageing and modifiable vascular risks, HIV/AIDS may be driving this burden with accompanying stigma, improved survival from the use of HAART and increasing neurologic/mental health complications. The determinants of cognitive dysfunction among PLWHA are yet poorly characterized. We investigated the correlates of cognitive dysfunction among PLWHA in Abeokuta, South West Nigeria.

**Method:**

This was a cross‐sectional study involving 352 persons living with HIV in three hospitals at Abeokuta, Southwestern Nigeria. The WHO stepwise interviewer‐administered questionnaire was used for data collection. Specific clinical information on HIV treatment status and traditional cardiovascular risk factors were assessed. Cognitive function was assessed using the IDEA score, functional outcome using IADL/Karnofovsky performance index, depression using GDS. Mann‐Whitney U and Kruskal Wallis tests were used to test the bivariate relationship between cognitive function and patient characteristics, thereby reporting the Median and interquartile range (IQR). Using quantile regression analysis, the independent determinants of cognitive performance in the study population were explored. The corresponding β coefficients (95% CI) were reported.

**Result:**

The mean (SD) age of the study population was 43.9±10.2 with predominant females (77.2%). A total 83 (23.6%) (1.7%) were hypertensive and and 6 study participants reported previous stroke. The median (IQR) IDEA score of the study population was 13(11, 13) and 4.8% (17) were cognitively impaired. Hypertension, alcohol use, low level of education and low income showed a significant bivariate relationship with cognitive dysfunction. In the quantile regression analysis, having adjusted for hypertension, alcohol use and income level, increasing level of education showed a dose‐response relationship with good cognitive performance with β (95% CI) of 2.0 (0.83 – 3.2) for primary education, 3.0 (1.83 – 4.2) for secondary education and 3.0 (1.7 – 4.3) for tertiary education.

**Conclusion:**

Among PLWHA, low level of education was independently association with cognitive performance. Population‐wide strategies targeted at improving educational attainment in SSA may achieve a dual objective of reducing the burden of communicable and non‐communicable disorders, like HIV and dementia.

